# Creating a Straight Conduit: The Pleural Closure Technique in Ivor Lewis Esophagectomy

**DOI:** 10.7759/cureus.91999

**Published:** 2025-09-10

**Authors:** Ethyn G Loreno, Mahnoor Zia, Ray Chihara, Warren C Naselsky, Min P Kim

**Affiliations:** 1 Surgery, Houston Methodist Hospital, Houston, USA

**Keywords:** anastomotic leak after esophageal cancer resection, gastric conduit, long term outcome, mie: minimally invasive esophagectomy, pleural closure, robotic surgical procedures

## Abstract

Ivor Lewis esophagectomy, a surgical procedure to treat esophageal cancer, requires gastric conduit creation with an intrathoracic anastomosis. Frequently encountered conduit complications include conduit redundancy, herniation, and dreaded complications of necrosis and anastomotic leak. We present the case of a 71-year-old male with esophageal cancer who underwent robot-assisted laparoscopic and thoracoscopic Ivor Lewis esophagectomy with total portal robotic linear stapled anastomosis with the omentum at the anastomosis and pleural closure. On postoperative imaging, the patient had a straight gastric conduit without redundancy or anastomotic leak. He had excellent long-term clinical outcomes without significant dysphagia and reflux. Pleural closure during robotic Ivor Lewis esophagectomy may aid in achieving excellent functional outcomes.

## Introduction

Surgical resection remains the primary curative treatment for esophageal cancer. Ivor Lewis esophagectomy is performed for distal esophageal and gastroesophageal junction (GEJ) cancer. It involves a two-stage procedure in which a gastric conduit is created in the abdomen and the anastomosis is created in the chest [1]. Potential conduit complications include conduit redundancy, herniation, necrosis, and anastomotic leak, which can lead to poor functional outcomes. In this report, we describe the technique of closing the pleura after total portal robotic linear stapled anastomosis, resulting in a straight conduit.

## Case presentation

The patient was a 71-year-old male with recurrent GEJ adenocarcinoma after declining initial surgical treatment. He had been initially diagnosed two years before presentation with stage III (cT2 N1 M0) esophageal adenocarcinoma at the GEJ and had a clinical complete response to seven cycles of chemotherapy (FOLFOX) followed by five weeks of radiation therapy. After treatment, the patient had declined surgery and elected to undergo continued surveillance. Six months before presentation, the patient had presented with dysphagia and underwent PET/CT and EGD with biopsy, which had confirmed cancer recurrence. The patient had a feeding jejunostomy placed and received neoadjuvant chemoimmunotherapy with FOLFOX and nivolumab, and subsequent PET/CT had shown persistent disease at the GEJ without distant metastatic disease.

The patient underwent robot-assisted, minimally invasive Ivor Lewis esophagectomy with total portal linear anastomosis with the omentum at the anastomosis [1] and subsequent closure of the pleura. Briefly, the procedure was started in the abdomen, with mobilization of the esophagus and placement of a Penrose around the esophagus, which was placed in the chest. Next, we mobilized the stomach, taking great care to preserve the gastroepiploic vessels, which would serve as the blood supply to the gastric conduit. We created a 3 cm gastric tube beginning at the incisura angularis toward the fundus, leaving approximately five venous drainages in the lesser curve of the stomach using six 45 mm robotic blue load staplers. We checked the perfusion of the conduit using ICG angiography (3 cc) to ensure adequate blood flow. The patient did not undergo a gastric drainage procedure.

**Figure 1:**
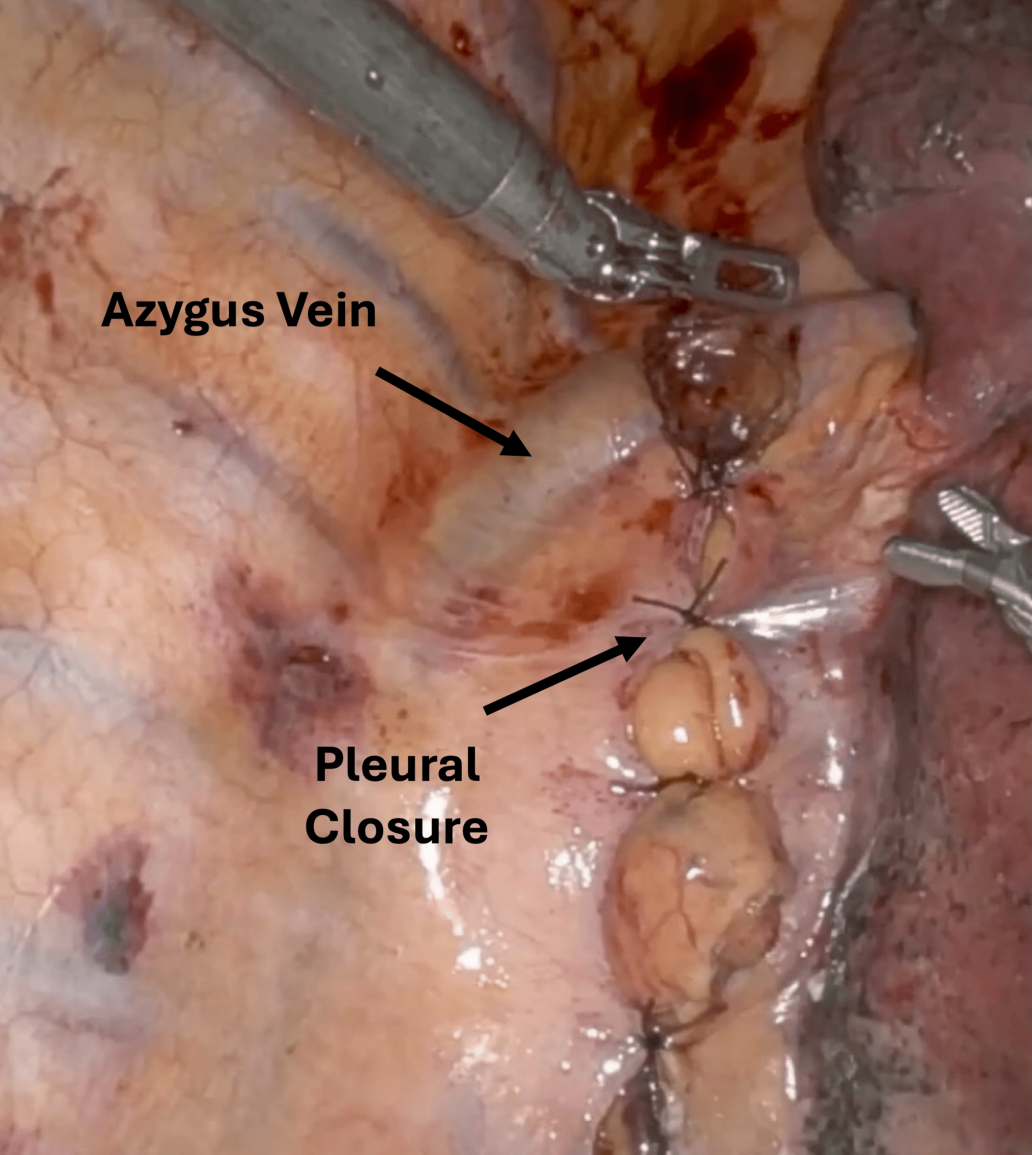
Photograph of pleural closure over the anastomosis and gastric conduit Closure started at the lower end of the azygus vein with 3-0 silk in an interrupted fashion down to the hiatus

Next, the patient was positioned on his side to allow entry into the right side of the chest. We divided the pleura over the Penrose, which was pulled out through the posterior inferior robotic port to provide traction on the esophagus. We then used a vessel sealer to divide the pleura over the esophagus up to the azygous vein. The azygus vein was mobilized and divided using a robotic vascular load stapler. Next, the esophagus was fully mobilized above the azygos vein to the distal esophagus, taking care to keep the pleura intact using a vessel sealer and bipolar. The esophagus was divided approximately 4 cm distal to the azygos vein, approximately 5 cm from the tumor. The proximal esophageal margin was sent to pathology, which was negative for malignancy. A total portal linear anastomosis was performed with closure of the common channel with a 3-0 absorbable V-loc and an additional horizontal mattress layer of 3-0 silk. We placed a small patch of the omentum over the anastomosis. Pleural closure was performed by bringing together the previously divided pleura over an intrathoracic conduit starting at the azygous vein. The pleura here is a thin tissue; thus, we carefully approximated it with 3-0 silk sutures approximately 3 cm apart (Figure 1, Video 1).

**Video 1:** Robot-assisted minimally invasive Ivor Lewis esophagectomy Video showing key parts of the robot-assisted minimally invasive Ivor Lewis esophagectomy, including creation of the conduit, intrathoracic esophagogastric anastomosis, and closure of the pleura

**Figure 2:**
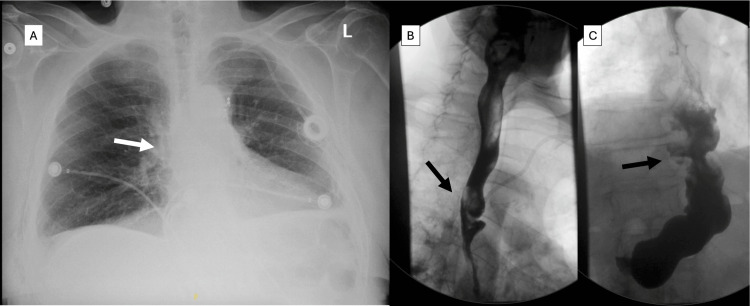
Imaging after esophagectomy Chest radiography showing no bulge in the right hilum (arrow, A). Esophagram shows a straight conduit at the anastomosis (arrow, B) and diaphragmatic hiatus (arrow, C)

The early postoperative course was uneventful. Postoperative chest X-ray (Figure 2A) and esophagram (Figures 2B-2C) demonstrated a relatively straight conduit and the absence of a leak. The patient’s pathology was stage II, ypT3N0 poorly differentiated adenocarcinoma with negative margins. At six weeks, the patient tolerated a regular diet, and eight months after surgery, the patient was able to tolerate liquids, soft food, bread, vegetables, and fruits without any difficulty. The patient had occasional difficulties eating meat. The patient stated that his heartburn symptoms were noticeable but not bothersome on Protonix, and he was satisfied with his condition. 

## Discussion

We performed robot-assisted minimally invasive esophagectomy, which is associated with a higher rate of R0 resection and retrieval of >20 lymph nodes, with lower rates of major complications, reinterventions, ICU admission, hospital stay, mortality, and 30-day readmission, compared to conventional minimally invasive esophagectomy [2]. Angeramo et al. showed that the overall morbidity of the robotic approach was 30%, which was significantly lower than that of the minimally invasive non-robotic approach (40%) (2). 

The overall incidence of anastomotic leak is approximately 14.2%, and the conduit necrosis rate is 2.7% [3]. Other complications include conduit hernia, which can occur in up to 19% of patients after esophagectomy [4], and conduit redundancy requiring surgery, which can occur in approximately 4% [5]. The overall functional outcome after surgery includes dysphagia, the incidence of which can be as high as 65% of cases [4], and reflux rate, which is as high as 69% [6]. Many of these conduit complications may potentially improve with pleural closure.

The placement of the anastomosis under the pleura was first described by Chassot et al. in their 1997 paper, which described this approach in 43 consecutive patients who underwent Ivor Lewis esophagectomy and for whom no leakage was demonstrated on the postoperative esophagram [7]. A recent study showed that complete mediastinal envelope closure during minimally invasive esophagectomy was associated with decreased rates of anastomotic leak (2% vs. 14.7%, p=0.007), postoperative pyloric dilation (15.6% vs. 32.4%, p=0.025), and delayed gastric emptying (6.1% vs. 20.6%, p=0.015), endorsing the benefit of additional structural support at the anastomosis [8]. Moreover, our patient had minimal dysphagia and good reflux control with pleural closure.

## Conclusions

Robotic Ivor Lewis esophagectomy may offer a promising approach to managing distal esophageal and GEJ cancers, with potential benefits including improved oncologic outcomes and reduced perioperative morbidity over the current standards of care. Despite these advances, postoperative complications, such as conduit hernia, anastomotic leaks, and poor functional outcomes, remain significant concerns. By incorporating pleural closure, our approach may enhance conduit alignment and decrease the complication rate after surgery. Further studies and long-term follow-up are needed to confirm the effectiveness of these aims and to optimize postoperative recovery.
